# Supplementary material for: YBX1 Regulates Satellite II RNA Loading into Small Extracellular Vesicles and Promotes the Senescent Phenotype
Source: Int J Mol Sci. 2023 Nov 16;24(22):16399. doi: 10.3390/ijms242216399 (PMC10671301; doi:10.3390/ijms242216399)
Supplement: Supplementary file 1 [file ijms-24-16399-s001.zip › ijms-2708899-supplementary.pdf]

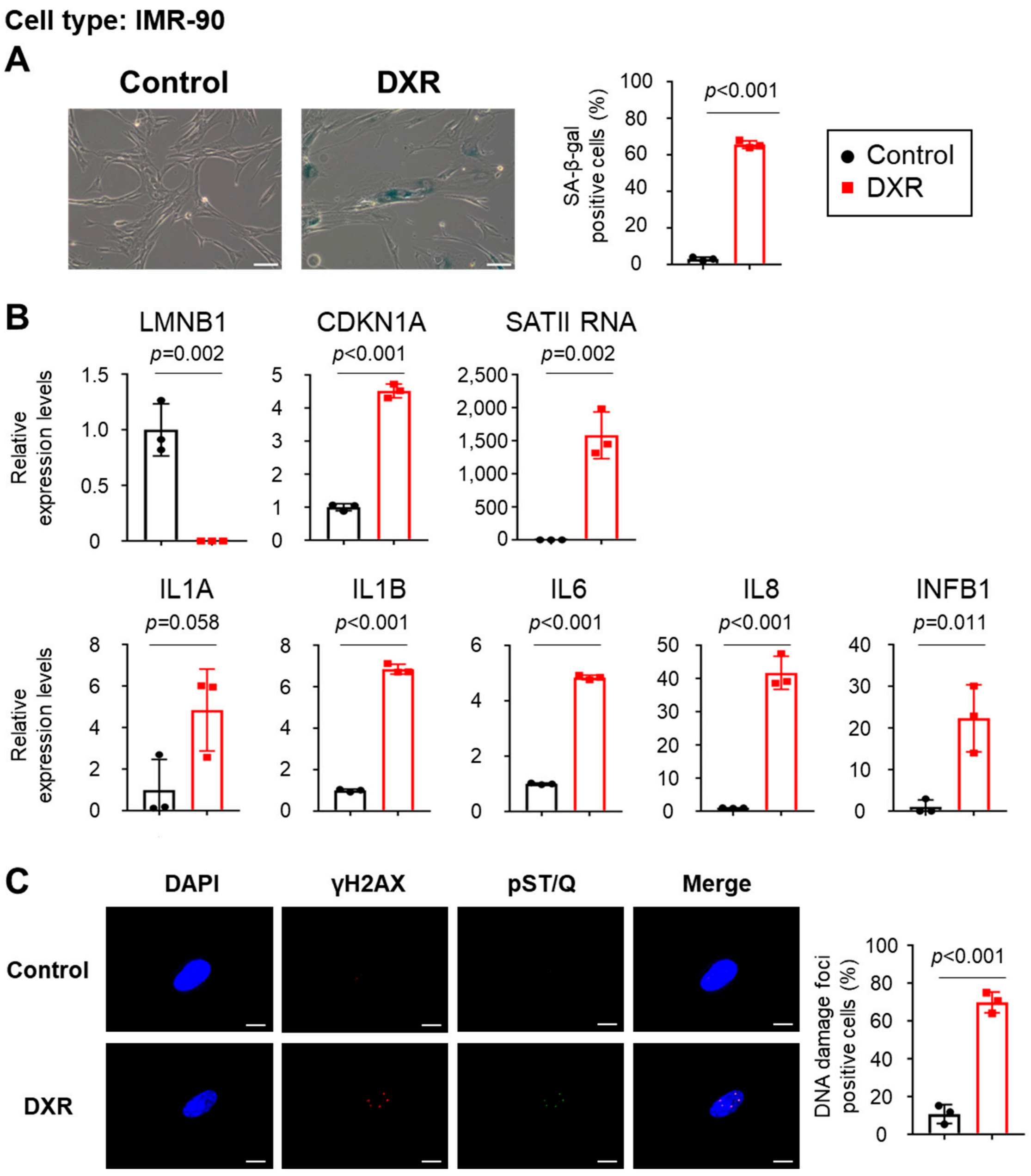

Figure S1. Cellular senescence was induced by doxorubicin (DXR) treatment in IMR-90 cells. (A) IMR-90 cells were treated with 200 ng/mL DXR for 11 days to induce cellular senescence. Senescence-associated β-galactosidase (SA-β-gal) staining of the cells before and after DXR treatment for 11 days. The bar graphs indicate the percentage of SA-β-gal–positive cells. Scale bar = 100 μm. (B) Relative RNA levels of *LMNB1*, *CDKN1A*, *IL1A*, *IL1B*, *IL6*, *IL8*, *INFB1*, and SATII RNA in control and irradiated cells by reverse transcription quantitative polymerase chain reaction. Relative quantitation data represent the mean ± standard deviation (SD) normalized to actin β. (C) Immunofluorescence staining of DNA damage response markers γH2AX (red), pST/Q (green), and 4′,6-diamidino-2-phenylindole (blue) in the cells before and after doxorubicin

addition. The bar graphs indicate the percentage of nuclei containing more than two positive foci for both γH2AX and pST/Q staining from at least 100 cells per condition for three independent experiments. Scale bar = 10 μm. Results represent the mean ± SD. *p*-Values were calculated by unpaired two-tailed Student's *t*-test in all panels.

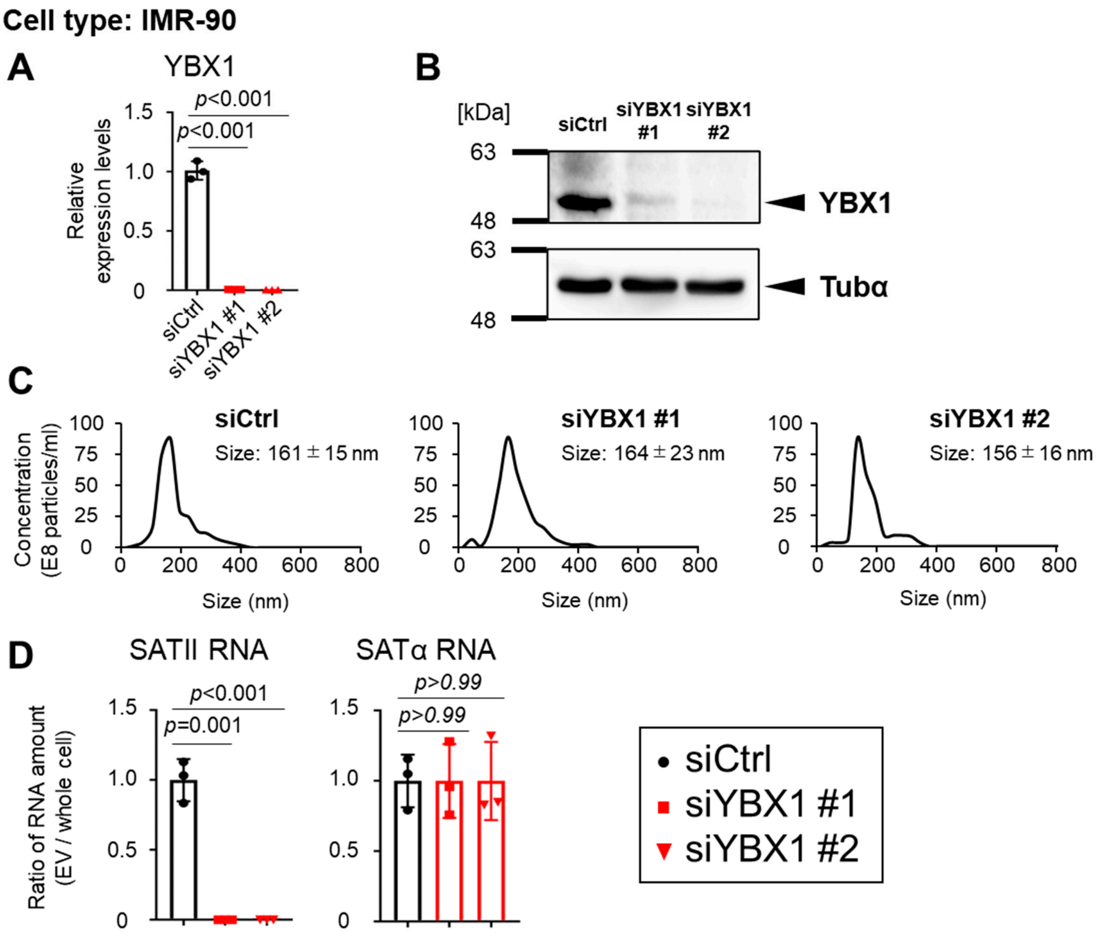

Figure S2. YBX1 knockdown reduced SATII RNA in sEVs released from IMR-90 cells. (A) Relative mRNA levels of *YBX1* in siCtrl cells and YBX1 knockdown IMR-90 cells were measured by reverse transcription quantitative polymerase chain reaction. Relative quantitation data represent the mean ± standard deviation (SD) normalized to actin β. (B) Western blot analysis of whole cell lysate of siCtrl and YBX1 knockdown IMR-90 cells. (C) Nanoparticle tracking analysis for quantitative measurement of sEVs collected from siCtrl or YBX1 knockdown cells. (D) Ratio of the amount of SATII RNA and SAT α RNA contained in sEVs divided by the amount contained in whole cells under treatment with YBX1 or control siRNA. Relative quantitation data represent the mean ± SD. *p*-Values were calculated by one-way analysis of variance with Dunnett's multiple comparisons test in panels A and E.
